# Biocompatibility and Antibacterial Effect of Ginger Fraction Loaded PLGA Microspheres Fabricated by Coaxial Electrospray

**DOI:** 10.3390/ma16051885

**Published:** 2023-02-24

**Authors:** Jung-Eun Park, Yu-Kyoung Kim, Seo-Young Kim, Ji-Bong Choi, Tae-Sung Bae, Yong-Seok Jang, Min-Ho Lee

**Affiliations:** 1 Department of Dental Biomaterials, Institute of Biodegradable Material, School of Dentistry, Jeonbuk National University, Jeon-ju 54896, Republic of Korea; 2 Institute of Oral Bioscience, School of Dentistry, Jeonbuk National University, Jeon-ju 54896, Republic of Korea

**Keywords:** electrospray, microencapsulation, poly(lactic-co-glycolic acid), ginger fraction, antibacterial activity, drug delivery

## Abstract

Various poly(lactic-co-glycolic acid) (PLGA) microspheres loaded with the ginger fraction were fabricated by controlling the electrospray parameters and their biocompatibility and antibacterial activity were identified in this study. The morphology of the microspheres was observed using scanning electron microscopy. The core-shell structures of the microparticles and the presence of ginger fraction in the microspheres were confirmed by fluorescence analysis using a confocal laser scanning microscopy system. In addition, the biocompatibility and antibacterial activity of PLGA microspheres loaded with ginger fraction were evaluated through a cytotoxicity test using osteoblast MC3T3-E1 cells and an antibacterial test using Streptococcus mutans and Streptococcus sanguinis, respectively. The optimum PLGA microspheres loaded with ginger fraction were fabricated under electrospray operational conditions with 3% PLGA concentration in solution, an applied voltage of 15.5 kV, a flow rate of 15 µL/min in the shell nozzle, and 3 µL/min in the core nozzle. The effectual antibacterial effect and enhanced biocompatibility were identified when a 3% ginger fraction in PLGA microspheres was loaded.

## 1. Introduction

Biodegradable polymer microspheres have been widely used in the field of medical drug delivery because they facilitate the delivery of drug molecules with various properties through multiple routes and have the advantage of controlling the low-dose loading of drugs and low bio-side effects [1,2]. Representative methods for producing micro- and nano-sized mixed multi-structured particles include batch emulsification [2,3], emulsification-curing [2,4], microemulsion [1,5], coacervation [6], microfluidic [1,7], spray drying [8], and high-voltage electrospray [9]. Traditional techniques, such as emulsification-curing and coacervation methods are time-consuming, resulting in low capture efficiency having problems such as the biotoxicity of residues and changes in chemical structure due to the use of organic solvents and surfactants. Therefore, it is difficult to apply these methods to drug delivery systems since the drug-loaded micro-nanoparticles have to stay in the body for the short/long term or be absorbed in the body gradually [1,2]. In order to overcome the limitations of these methods, the electrospray (ES) method has been introduced as an alternative method that can produce the desired micro and nanoparticles [10].

The high-voltage electrospray method can physically control the size of the microspheres and the amount of drug loaded in the carrier while minimizing the use of toxic and genetically unverified organic solvents. In addition, micro/nanoparticles fabricated by the ES method have excellent monodispersity, easy adjustment of drug loading, excellent capture efficiency, and simple operation [11,12]. In the electrospray method, when a high voltage is applied between the spray nozzle and collector, ionized droplets are formed, which are separated from the solution surface at the tip of the nozzle where the material is sprayed. In other words, it is a method to obtain polymer spheres that have fallen into the collector by electrostatic force and gravity acting on the material simultaneously. The size of the sphere is immediately reduced by evaporation of the solvent after the droplet falls into the collector solution and undergoes a separation process by Coulomb fission [13,14]. The electrospray process is affected by various parameters, such as the flow rate of the solution in the nozzle, the concentration of the solution, conductivity and density of the solution, applied voltage, and distance between the nozzle and the integrated plate. Coaxial electrospray (CES), a microencapsulation technology, can produce uniform multi-shell capsules in a relatively short time by injecting different materials into the outer and inner nozzles using the ES method with multiple coaxial nozzles. It is used in fields such as food, cosmetics, and pharmaceuticals, to protect the substance inside the capsule or induce the release of the desired drug amount. In this study, ginger extract, as a natural antibacterial substance, was injected into a biodegradable polymer shell using the dual co-axial electro spraying method to develop microcapsules for the application in oral prophylaxis, which requires biocompatibility and antibacterial properties.

Poly(lactic-co-glycolic acid) (PLGA), a widely used synthetic biodegradable polymer, is synthesized by randomly polymerizing two monomers, lactic acid and glycolic acid. Since the molecular weight of the polymer and the ratio of lactic acid and glycolic acid can change the mechanical strength and biodegradation rate, it is necessary to appropriately select the molecular weight and monomer ratio according to the application. PLGA is biocompatible because it is hydrolyzed back to lactic acid and glycolic acid in vivo, which are metabolized to water and carbon dioxide (CO_2_) through the tricarboxylic acid cycle and discharged from the lung [15,16].

Ginger (*Zingiber officinale* Rosc.), selected as a natural product in this study, was the rhizome of a subtropical perennial herbaceous plant belonging to the family Zingiberace-ae [17]. Physiologically active components of ginger have antibacterial and anti-inflammatory activity, serum cholesterol lowering function, and antioxidant effects [18,19]. Ginger contains gingerol, shogaol, zingerone, citral, and zingeperone, which have a unique stimulant taste and are used as a disinfectant and therapeutic agent [20]. The raffinose component in ginger has effective antibacterial activity against specific bacteria such as *Streptococcus mutans* and *Porphyromonas gingivalis* in teeth, inhibiting the formation of bacterial biofilms that can cause dental caries and periodontitis by bacterial infection [21,22].

The purpose of this study was to establish optimum electrospray conditions for the fabrication of PLGA microcapsules loaded with ginger fraction and identify their biocompatibility and antibacterial effects against oral bacteria. PLGA microcapsules were first developed using a coaxial electro spraying system. Coaxial electro spraying can spray different materials from the two nozzles. PLGA dissolved in an organic solvent and ginger extract has different viscosities, volatilities, and densities. Therefore, it is crucial to determine the optimum parameters, such as the concentration and viscosity of the material, flow rates of materials in the core and shell nozzles and applied voltage for successful encapsulation of the ginger fraction in the PLGA shell. Various PLGA microcapsules loaded with ginger extract were obtained by controlling these conditions. The shape of the microcapsules and the presence of the ginger fraction loaded in the microcapsule were confirmed using scanning electron microscopy (SEM) and fluorescence analysis. Antibacterial activity was evaluated using two oral bacteria, *S. mutans* and *S. sanguinis.* Biocompatibility was investigated using a cytocompatibility test with osteoblast cells.

## 2. Material and Method 

### 2.1. Materials

PLGA (LA:GA/75:25), polyvinyl alcohol, acetonitrile (ACN), fluorescein 5(6)-isothiocyanate (FITC), and Rhodamine B (RhB) were purchased from Sigma-Aldrich (St. Louis, MO, USA). Dichloromethane (DCM) was purchased from SHOWA (Tokyo, Japan). The ginger fraction was obtained from the National Institute of Agricultural Science of the Rural Development Administration, Republic of Korea. The manufacturing method of the ginger fraction is as follows: the hot-air dried ginger powder was mixed with 70% ethanol containing 10% *w*/*v* citric acid. It was then extracted with a stirrer for 4 h at 60 °C before filtration and concentration. The concentrate was dissolved in water and fractionated using *n*-hexane (C_6_H_14_) and chloroform. The fractionated solution was freeze-dried to obtain the final ginger fraction.

### 2.2. Preparation of PLGA Capsules Using the Coaxial Electrospraying Method

PLGA microspheres loaded with ginger fractions were prepared as follows: The ginger fraction solution entering the core nozzle was prepared by dissolving the ginger fraction obtained from the National Institute of Agricultural Science of the Rural Development Administration in a solvent mixed with DW and ACN 50:50. 

**Figure 1:**
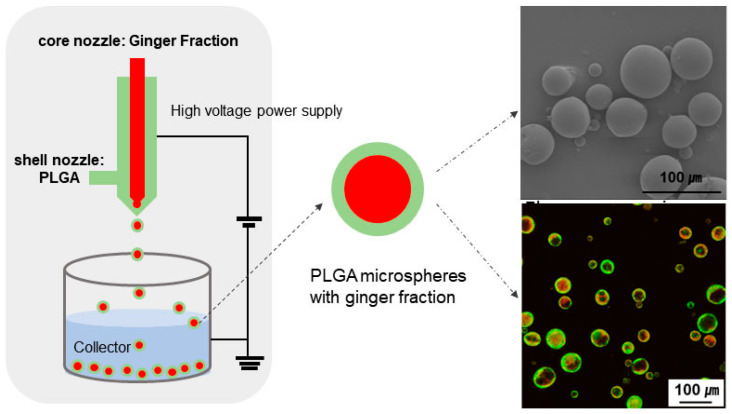
Schematic of coaxial electro spraying devices.

Subsequently, 3% PLGA solution entering the shell nozzle was prepared by dissolving PLGA in a solvent mixture of ACN and dichloromethane, 2:1. The prepared 0.1% ginger fraction solution and 3% PLGA solution were placed in a core nozzle and a shell nozzle, respectively. The core and shell nozzles used were 30 G (outer diameter: 0.32 mm, inner diameter: 0.14 mm) and 22 G (outer diameter: 0.7 mm, inner diameter: 0.4 mm) needles, respectively. The coaxial needle and collector were connected to the anode and cathode of the high-voltage DC power source (NanoNC, Seoul, Korea). The vertical distance between the coaxial needle and collector was fixed at 150 mm. The 1% polyvinyl alcohol solution was used as the collector. The coaxial electrospray test method is schematically shown in Figure 1, and the experimental conditions for coaxial electro spraying are shown in Table 1.

### 2.3. Characteristic of Microspheres 

The morphology of the particles was assessed using scanning electron microscope (SEM; SU-70, HITACHI, Tokyo, Japan). The accelerating voltage used was 5 kV, and the current was 5.0 µA. The core-shell structures of the microparticles were evaluated using a confocal laser scanning microscopy system (C2+, Nikon, Tokyo, Japan). For fluorescence color display, 1 mg/mL FITC was added to the PLGA solution, and 1 mg/mL RhB was added to the 0.1% ginger fraction solution. The FITC containing PLGA were obtained with excitation wavelengths of 503 nm and emission wavelengths 553 nm. The RhB containing ginger fraction were obtained with excitation and emission wavelengths of 663 nm and over 720 nm, respectively. Fourier-transform infrared (FT−IR) spectroscopy (Spectrum GX, Perkin Elmer, Waltham, MA, USA) was performed at the Center for University-wide Research Facilities (CURF) of Jeonbuk National University to determine the chemical bonding properties of PLGA microspheres with ginger fraction in the range 500–4000 cm^−1^.

### 2.4. Immersion Test

15 mg of PLGA microspheres loading with ginger fraction was incubated in 5 mL Phosphate-Buffered Saline (PBS) at 37 °C, in a constant-temperature shaking incubator for 15 days. The morphology of PLGA microspheres loading with ginger fraction was observed by SEM.

### 2.5. In Vitro Test

In the antibacterial and cell tests, the materials were tested in three groups of PLGA microspheres loaded with 0%, 0.1% and 0.3% ginger fractions. As the selection criterion for the concentration of the ginger fraction, minimum inhibitory concentration of 0.1%, which showed antibacterial properties, was selected in the pure ginger fraction without PLGA microcapsules.

#### 2.5.1. Antibacterial Test

##### Bacteria Culture

*Streptococcus mutans* and *S. sanguinis* were selected as the test bacterial strains. All bacterial strains were procured from the Korean Collection for Type Cultures (KCTC) and precultured on sheep-blood agar plates (BAP; ASAN Pharmaceutical, Seoul, Korea) for bacterial growth assays. The strains were grown in an incubator for 48 h at 37 °C in the presence of 5% CO_2_. A standardized suspension of all bacterial strains was diluted with brain–heart infusion (BHI; Difco, Becton Dickinson and Company, Sparks, MD, USA) broth to obtain a bacterial count of 1.5 × 10^7^ colony-forming units/mL.

##### Antibacterial Activity Test

The antibacterial activity of ginger extract in PLGA microspheres was evaluated. PLGA microspheres loaded with ginger extract were eluted in BHI for 7 d. Bacteria were cultured in the eluate to conduct the test. A standardized suspension (100 µL) of each strain was added to 100 µL of the diluted agent. After incubation in a 5% CO_2_ incubator at 37 °C for 24 h, absorbance was measured using an enzyme-linked immunosorbent assay microplate reader (EMax PRECISION MICROPLATE READER; Molecular Devices, Sunnyvale, CA, USA) at a wavelength of 600 nm. Bacterial growth was calculated as follows:

Viability (%) = experimental Absorbance/Control Absorbance × 100

#### 2.5.2. Cell Test

##### Cell Culture 

The cells used in this study were Mouse Osteoblastic (MC3T3-E1) Cell Line (ATTC; American Type Culture Collection, Manassas, VA, USA). The culture solution was prepared by adding 10% fetal bovine serum (FBS; Gibco Co., New York, NY, USA) containing penicillin as a nutrient to the α-MEM medium (Gibco Co., New York, NY, USA). PLGA microspheres were eluted with the cell culture medium for 7 d. The cells were then cultured in the eluted medium. The cells were cultured in a 5% CO_2_ incubator (3111; Thermo Electron Corporation, Waltham, MA, USA) at 37 °C.

##### Cell Morphology and Proliferation 

MC3T3-E1 cells were cultured in 48-well plates at a density of 1 × 10^5^ cells/mL for 1 and 3 days. Following cell culture, the medium was removed, and the cells were washed with PBS. A fixative (3% formaldehyde + 0.2% glutaraldehyde) was added and maintained for 10 min before removal. The cells were then washed with PBS and fixed. The surviving cells were stained with 0.3% crystal violet dye. Cell morphology was observed with an optical microscope (DM2500, Leica, Germany). 

Cell proliferation was examined using a water-soluble tetrazolium salt. After days 1 and 3, the medium was removed. A solution prepared by mixing the cell counting kit-8 (CCK-8; Enzo Life Science Inc., New York, NY, USA) reagent and the α-MEM medium was dispensed in 400 µL/well and reacted with the cells in a 5% CO_2_ incubator at 37 °C. After 1 h, they were injected in 100 µL units into a 96-well plate. The absorbance was measured at 450 nm using an enzyme-linked immunosorbent assay reader (Molecular Devices, San Jose, CA, USA). 

#### 2.5.3. Statistical Analysis 

All experiments were performed five times. Statistical analyses were conducted using one-way analysis of variance. Data were expressed as the mean ± SD. A p-value of less than 0.05 was considered to be significant (* *p* < 0.05). When there was no significant difference, it was marked as ns (no statistical significance).

## 3. Results 

**Figure 2:**
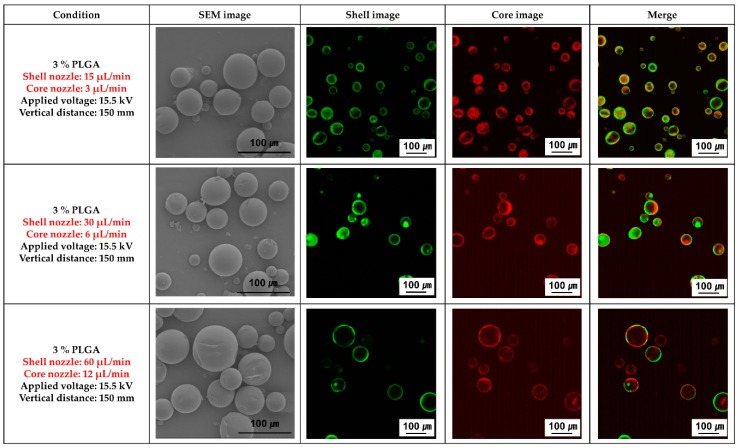
Scanning electron microscopy (SEM) and fluorescence analysis images of poly(lactic-co-glycolic acid) (PLGA) microparticles loaded with ginger fraction according to the flow rate of the core and the shell nozzles.

Figure 2 shows the SEM morphology of the PLGA microcapsules, and the fluorescence images obtained by varying the flow rates of the solutions in the core and shell nozzles of the coaxial electrospray. It was confirmed that the PLGA spheres had a uniform size distribution regardless of the flow rate of the solutions in the core and shell nozzles. However, the size of the spheres increased as the flow rate of the solutions in both nozzles increased. In addition, the best uniform and stable loading of the ginger fraction in the PLGA sphere was confirmed when the flow rates of the solutions in the shell and core nozzles were 15 µL/min and 3 µL/min, respectively.

**Figure 3:**
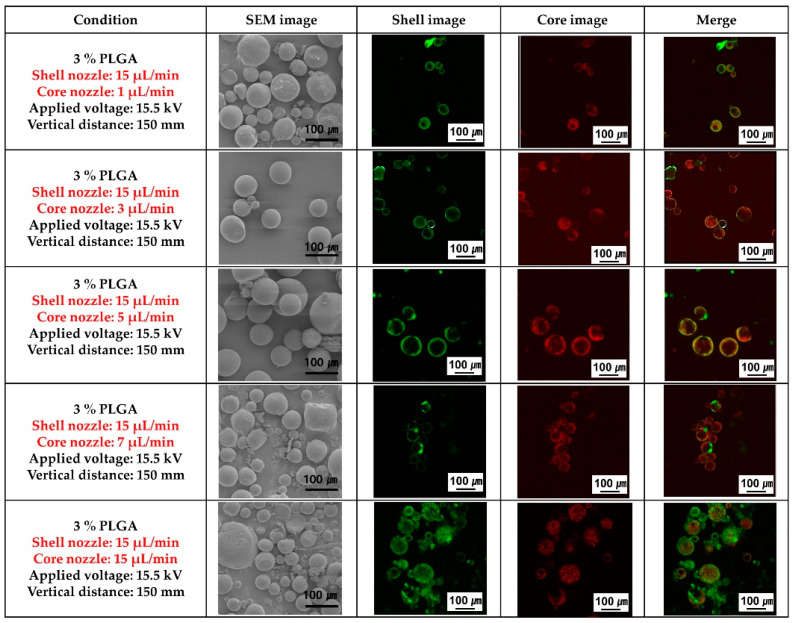
Scanning electron microscopy (SEM) and fluorescence analysis images of poly(lactic-co-glycolic acid) (PLGA) microparticles loaded with ginger fraction according to the flow rate of the core nozzle.

Figure 3 shows the SEM morphology of the PLGA microcapsules and fluorescence images to confirm the presence of the ginger fraction in the PLGA microcapsules according to the flow rate of the ginger fraction solution in the core nozzle, while the flow rate of the PLGA solution in the shell nozzle was fixed at 15 μL/min during coaxial electro spraying. PLGA spheres with non-uniform size distributions were observed when the flow rate of the ginger fraction solution in the core nozzle was over 7 μL/min or under 1 μL/min. PLGA spheres with uniform size and stable loading of the ginger fraction solution in PLGA spheres were observed when the flow rate of the ginger fraction solution in the core was 5 μL/min.

**Figure 4:**
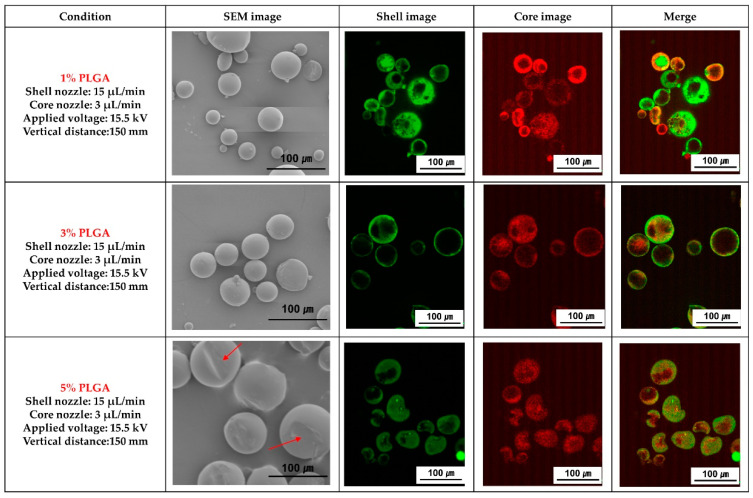
Scanning electron microscopy (SEM) and fluorescence analysis images of poly(lactic-co-glycolic acid) (PLGA) microparticles loaded with ginger fraction according to PLGA concentration.

Figure 4 shows SEM and fluorescence images of the PLGA microcapsules fabricated in PLGA solutions with different concentrations by coaxial electro spraying. The 1% and 3% PLGA microcapsules exhibited a spherical shape but the 5% PLGA microcapsules showed a marginally distorted spherical shape, as shown in SEM and fluorescence images. It was confirmed that the ginger fraction was loaded in the PLGA microcapsules.

**Figure 5:**
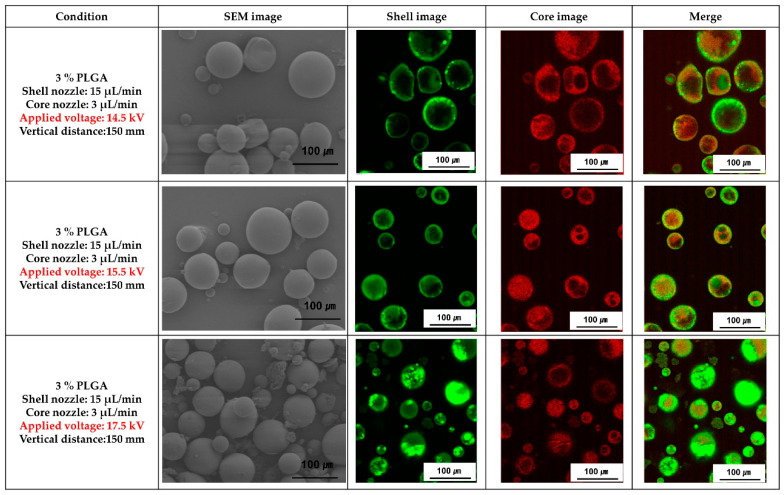
SEM and fluorescence analysis images of PLGA microparticles loaded with ginger fraction according to the applied voltage.

Figure 5 shows SEM and fluorescence images of the PLGA microcapsules fabricated with various voltages applied during coaxial electro spraying. When the applied voltage was 14.5 kV, the microcapsules were out of spherical shape, but the microcapsules manufactured with an applied voltage of 15.5 kV and 17.5 kV showed a spherical shape. In addition, the PLGA microcapsules fabricated with the applied voltage of 17.5 kV had non-uniform size distribution, and smaller microcapsules were observed compared with those fabricated at 15.5 kV. As shown in fluorescence images, the ginger fraction was loaded in all PLGA microcapsules.

**Figure 6:**
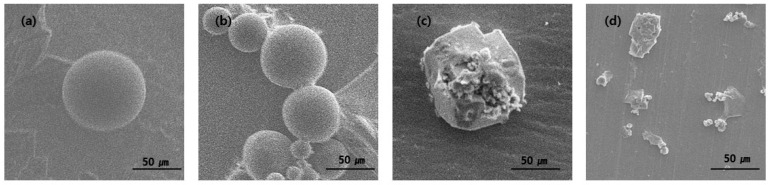
SEM image of PLGA microparticles loaded with ginger fraction according to the immersion time (**a**) 1 day, (**b**) 5 days, (**c**) 10 days and (**d**) 15 days.

As shown in Figure 6, the morphology of PLGA microspheres loading with ginger fraction changed significantly with increased immersion time. After immersion for 1 and 5 days, the shape of the capsule maintains a spherical shape. After 10 days of immersion, the spherical shape begins to unravel. After immersion for 15 days, no spherical shape was observed. The initial microsphere degradation was observed after 10 days of incubation. Subsequently, the structure is loose with the degradation of PLGA microspheres loading with ginger fraction.

**Figure 7:**
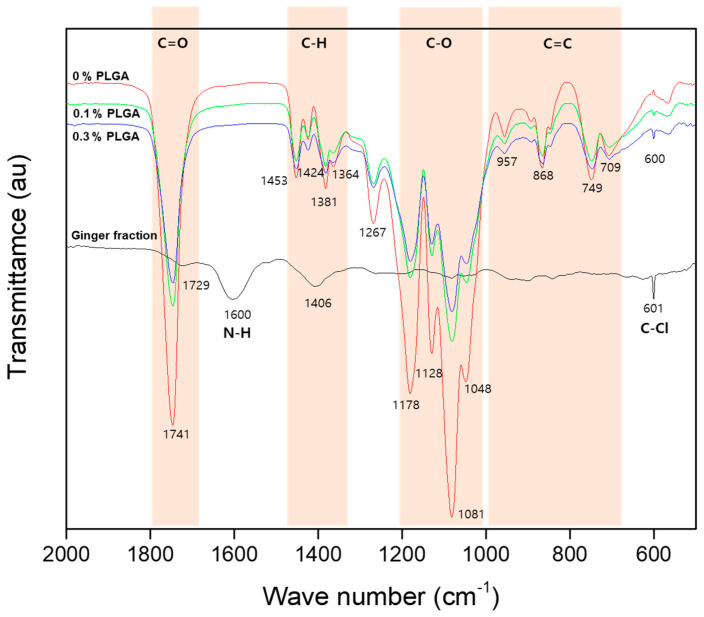
The IR spectrum of ginger fraction, 0% PLGA, 0.1% PLGA and 0.3% PLGA.

Figure 7 shows the FT-IR spectrum of the ginger fraction and PLGA microcapsules loaded with the ginger fraction. The ginger fraction spectrum shows the peaks of C=O stretching vibrations (1700–1800 cm^−1^), the N-H bending vibration (1580–1650 cm^−1^), and the C-Cl stretching vibrations (550–850 cm^−1^). The PLGA spectrum show the peaks of the C=O stretching vibrations (1700–1800 cm^−1^), the C-H bending vibration (1365–1465 cm^−1^), the C–O stretching vibrations (1050–1250 cm^−1^), and the C=C bending (650–1000 cm^−1^). The PLGA microcapsules loaded with the ginger fraction show the same peak as 0% PLGA, and the peak of C-Cl stretching vibrations (550–850 cm^−1^) was observed. In addition, as the content of ginger increased, the C-Cl stretching vibrations (550–850 cm^−1^) peak appeared stronger.

**Figure 8:**
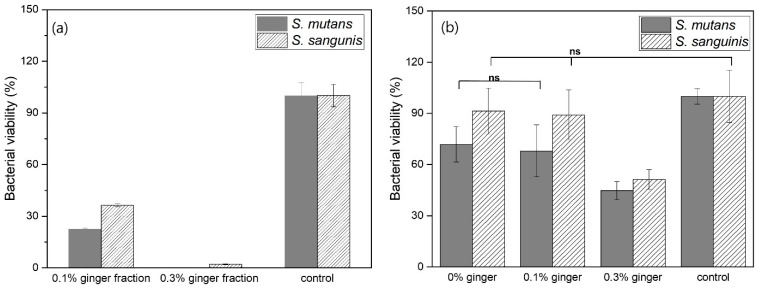
Bacterial viability of (**a**) pure ginger fraction concentration and (**b**) PLGA microspheres loading with the ginger fraction content to *Streptococcus mutans* and *Streptococcus sanguinis* after 24 h (ns: no statistical significance, *p* > 0.05).

Figure 8a shows the bacterial viability of pure ginger fractions without PLGA capsules. In the results of *S. mutans*, the bacterial viability of the 0.1% ginger fraction was about 22%, and the bacterial viability of the 0.3% ginger fraction did not appear. In addition, in the results of *S. sanguinis*, the bacterial viability of 0.1% ginger fraction and 0.3% ginger fraction was about 35% and about 2%, respectively. In the results of *S. mutans* and *S. sanguinis*, the bacterial viability of the PLGA microspheres loaded with 0.1% ginger fraction was about 67% and 89%, respectively, and the bacterial viability of the PLGA microspheres group loaded with 0.3% ginger fraction were about 45% and about 50%, respectively. When comparing the bacterial viability of the pure ginger fraction without PLGA capsule and the ginger fraction loaded into PLGA capsule, the bacterial viability was lower in the pure ginger fraction without PLGA capsule.

**Figure 9:**
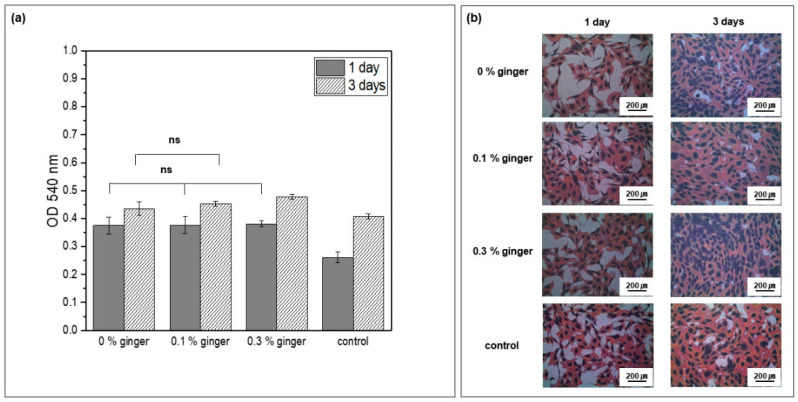
(**a**) Proliferation and (**b**) cell morphology of MC3T3-E1 cells cultured after days 1 and 3 (ns, not statistically significant, *p* > 0.05).

To identify the biocompatibility of PLGA microcapsules, including the ginger fraction, MC3T3-E1 cells were cultured for days 1 and 3 in culture media in which PLGA microcapsules fabricated with different concentrations of ginger fraction were dispersed. Cell proliferation was confirmed. As shown in Figure 9a, there was no statistically significant difference between the PLGA microcapsule groups on day 1 (ns, *p >* 0.05). However, it was confirmed that cell proliferation in the PLGA microcapsule groups was improved compared with that in the control group. On the third day of cell culture, the highest cell proliferation was confirmed in the PLGA microcapsule group loaded with 0.3% ginger fraction. There was no statistically significant difference between PLGA microcapsule groups loaded with 0% and 0.1% ginger fractions. In addition, the groups containing PLGA microcapsules showed improved cell proliferation compared with the control group. As a result of checking the cell morphology in Figure 9b, all groups showed a cytoplasm developed around the nucleus of the cells. More cell nuclei were observed on the third day than on the first day.

## 4. Discussion

Biodegradable microspheres have been widely used in the medical field due to their ability to deliver drug molecules with various properties through multiple routes, low doses, and lower side effects [23]. The electrospray method used to produce microparticles is becoming increasingly popular and has the advantage of controlling the size and shape of the resulting particles. Particle size and morphology are key factors that control the rate of microsphere disintegration and drug diffusion.

In this study, we developed PLGA microspheres loaded with ginger fractions using a coaxial electrospray system. By controlling the parameters of the coaxial electrospray system, we aimed to establish optimal conditions for the encapsulation of the ginger fraction in PLGA microcapsules.

Therefore, in this study, the shape of the PLGA microcapsules loaded with the ginger fraction was confirmed according to the change in flow rate of the shell and core nozzles of the coaxial electrospray (Figure 2). The PLGA capsule was spherical, regardless of the flow rate of the core and shell nozzles. It was confirmed that the size of the capsule increased with the flow rate of the nozzle. The inner and outer flow rates of coaxial electro spraying play a crucial role in cone jet stability and droplet size. Mei et al. [24] found that stable core-shell structures can be formed for selected inner/outer liquid combinations under specific flow conditions. In a previous study, it was observed that droplet size decreased with a decreasing flow rate, which can be explained by the smaller electrical force required to overtake the hydrodynamic force at low flow rates [25].

Based on the results shown in Figure 2, the flow rate of the shell nozzle of the coaxial electrospray was fixed at 15 μL/min. The shape change of the PLGA microcapsules loaded with the ginger fraction was confirmed as the flow rate of the core nozzle changed (Figure 3). As a result, as the flow rate of the core nozzle increased to more than 7 μL/min, PLGA comprising fine particles was observed. In addition, when the flow rate of the core nozzle was 1 μL/min, the ginger fraction was not clearly observed. Because coaxial electro spraying involves cohesive interactions between multiple phases, maintaining an appropriate equilibrium between these phases is a key factor for the successful production of a core-shell structure. Torza and Mason established the engulfing conditions for two immiscible materials in shear and electric fields [26]. According to their analysis, the following three situations (as shown in Figure 5) may occur if phase 1 (core material) and phase 3 (shell material) are brought into contact in phase 2 (air for spraying and liquid for collection): complete engulfing, partial engulfing, and non-engulfing. To ensure the successful formation of a core-shell structure, the inner and outer liquids must satisfy the complete engulfing condition [26]. 

Changing the concentration of a polymer solution can change the particle size [27]. The concentration of PLGA is an important parameter in the entanglement region, which controls the electrospray process and directs the formation of fibers or particles [12]. In this study, PLGA microcapsules loaded with ginger fractions were fabricated using different concentrations of the polymer solution (Figure 4). The shape of the microcapsules was not perfectly spherical as the concentration of the polymer solution increased. The size and shape of the electro sprayed particles can be controlled by the diffusion of polymer molecules in the liquid solvent and the evaporation rate of the solvent [28]. Xie et al. reported that as the PLGA concentration increased, the diffusion rate of polymer chains decreased, and the evaporation rate of the solvent in the fabrication of polymeric particles using electrohydrodynamic spraying could form a highly concentrated PLGA on the surface of the droplets. In addition, this contributed to producing a larger shell diameter, leading to larger final particle sizes [29]. In a similar study, higher concentrations of PCL produced larger particles owing to reduced electrical conductivity with an increase in surface tension and viscosity [30].

The production of nanoparticles using electrospray is related to the droplet’s surface tension and the electrostatic forces inside the droplets. As the voltage increases, the meniscus of the liquid ejected across the emitter (metallic capillary) tip starts to stretch. Its shape is deformed, such as a cone or jet, and then emitted into droplets. During emission, the solvent evaporates from the surface of the droplet, reducing its size and increasing charge density. This causes a high repulsive force to accumulate inside each droplet, which disperses smaller droplets from the microscale to the nanoscale. This process continues until the charge dissipates after the droplet reaches the precipitator [31]. In this study, PLGA microcapsules loaded with the ginger fraction were fabricated by varying the applied voltage (Figure 5). As a result, it was difficult to obtain microcapsules with a perfectly spherical shape if a sufficient voltage was not applied.

In the CES technology, core-shell microparticles (MPs) are fabricated using two different solutions (drug and polymer) that flow through a coaxial syringe under the influence of an electric field [32]. Loxcertales et al. [33] introduced CES technology in 2002 and demonstrated the fabrication of dispersing single capsules using two immiscible liquids. Subsequently, several studies have been conducted to improve the quality and productivity of this technology. The properties and drug release profile of particles produced by the electro spraying method is affected by many parameters, such as solvent properties, voltage, flow rate, syringe-to-collector distance, drug/polymer ratio, syringe gauge, and external conditions [34,35]. While electro spraying is advantageous for engineering various particle surfaces with specific properties, it is necessary to guarantee reproducibility in fabrication. The optimization procedure of electrospray methodology is complex and difficult because numerous parameters are interactive. Therefore, fabrication conditions is established according to the required area. 

As the antibacterial activity of plants and herbs is generated by phytochemical compounds, phenolic compounds, and essential oils, many plant extracts show activity against microbial strains and substitutability for drug substitution [33]. Since the raffinose component in ginger inhibits the formation of a bacterial biofilm that causes tooth erosion or bacterial infection, it is effective in the prevention of dental caries on the tooth surface and antibacterial activity against specific bacteria in the oral cavity, such as *S. mutans* and *P. gingivalis* [21]. In this study, the antibacterial activity of PLGA microparticles loaded with ginger fraction against the oral bacteria *S. mutans* and *S. sanguinis* was confirmed (Figure 8). The PLGA microparticles loading 0.3% ginger fraction exhibited a growth inhibitory effect on both bacteria (Figure 8). Ginger-essential oil has been reported to have an inhibitory effect against *S. mutans* and the ability to disrupt biofilm formation; it is also effective against other oral pathogens [36,37]. Phytochemical compounds from ginger extract showed potential as antimicrobial agents for the treatment of infections in the oral cavity.

The surface of microspheres plays a vital role in cell adhesion and growth. To confirm the biocompatibility of PLGA microspheres loaded with the ginger fraction, the cytotoxicity of MC3T3-E1 cells was evaluated (Figure 9). After one day of cell culture, it was confirmed that cell proliferation was improved in the PLGA microspheres with and without the ginger fraction compared with the control group. In addition, the cells after three days of culture were most effectively proliferated in PLGA microspheres loading 0.3% ginger fraction. PLGA has an advantage in the promotion of osteosis owing to its three-dimensional porous structure similar to human cancellous bone [38]. In addition, PLGA has been approved by the FDA owing to its excellent stability, biocompatibility, and biodegradability, and is used as a material for various purposes in the human body. It is biodegradable and is degraded and absorbed by the human body through hydrolysis [39]. Tiep et al. [40] reported that there was no significant difference in the survival of ins-1 cells when PLGA microspheres loaded with quercetin, which has anti-inflammatory and antibacterial effects, were compared with those loaded without quercetin.

The limitation of this study is that although the loading was confirmed through FT-IR and fluorescence analysis, quantitative analysis of the loading efficiency of the ginger fraction in the PLGA microcapsule through high performance liquid chromatography (HPLC) analysis was not performed. Therefore, additional research is required to systematically verify the effect of antibacterial activity according to the loaded amount of ginger fraction in PLGA microcapsule.

## 5. Conclusions

To summarize, various PLGA microspheres loaded with ginger fractions were fabricated using controlling the electrospray parameters, such as the concentration of the material, the flow rate of the core nozzle and shell nozzle, and the applied voltage, to determine the optimal fabrication conditions. The optimum PLGA microspheres loaded with ginger fraction were fabricated under electrospray operational conditions with 3% PLGA concentration in solution, an applied voltage of 15.5 kV, a flow rate of 15 μL/min in the shell nozzle, and 3 μL/min in the core nozzle, respectively. The improved biocompatibility and antibacterial activity of the PLGA microspheres loaded with ginger fractions were confirmed. In particular, PLGA microspheres containing 0.3% ginger fraction inhibited bacterial growth. In addition, PLGA microspheres loaded with 0.3% ginger fraction showed improved cell proliferation.

Therefore, we fabricated PLGA microspheres loaded with ginger fractions with improved biocompatibility and antibacterial effect, which provided a wide range of applications in the dental field.

## Figures and Tables

**Table 1:** Experimental conditions for coaxial electro spraying.

Flow Rate [µL/min] Shell Nozzle:Core Nozzle	PLGA Concentration [%]	Applied Voltage [kV]	Vertical Distance [mm]
60:12	3	15.5	150
30:6	3	15.5	
15:1	3	15.5	
15:3	1	15.5	
	3	14.5	
		15.5	
		17.5	
	5	15.5	
15:5	3	15.5	
15:7	3	15.5	
15:15	3	15.5	

## Data Availability

Data available on request due to restrictions eg privacy or ethical.
